# Sutureless Approach for Gastroschisis Patients in Palestine

**DOI:** 10.1155/2020/8732781

**Published:** 2020-08-22

**Authors:** Yousef S. Abuzneid, Sadi A. Abukhalaf, Duha Rabi, Abdelrahman Rabee, Safwan Mashhour, Radwan Abukarsh

**Affiliations:** ^1^Al-Quds University Faculty of Medicine, Jerusalem, State of Palestine; ^2^Palestine Red Crescent Society Hospital, Hebron, State of Palestine

## Abstract

Gastroschisis is a ventral abdominal wall congenital defect with bowel herniation outside the abdominal cavity. Gastroschisis traditional management is the primary operative closure surgery (POCS), but the sutureless silo approach (SSA), a novel alternative, gains wide acceptance in the developed countries and across nations. This study describes the first-ever gastroschisis patient managed with the sutureless silo approach in Palestine. In addition, we shall use this case as the very first nucleus for the upcoming gastroschisis management in our referral hospital because the SSA yields a reduced hospital stay which is fundamental to our institution due to the limited number of beds and lower management costs to the hospital and families.

## 1. Introduction

Gastroschisis describes a birth defect of bowel evisceration outside the abdomen through a right-sided periumbilical abdominal wall defect [1]. The condition affects about 2 to 4 per 10,000 live births with male predominance, and its rate appears to be increasing [2]. Management typically involves fascia and skin closure. The conventional primary operative closure surgery (POCS) is performed immediately after birth by closure of the defect with sutures due to the provided evidence of the favorable outcomes [3].

In case of large defects with a small abdominal cavity or increased abdominal hypertension, the exposed organs may be contained with an artificial pouch or silo and slowly get moved back into the abdominal cavity followed by a sutured closure—delayed primary surgery or staged silo closure [4, 5]. However, these approaches have the disadvantages of requiring prolonged intubation and mechanical ventilation, narcotic analgesic use, ileus formation, prolonged hospital stay, and subsequently significant financial burden on both hospitals and families [6, 7].

In 2004, Sandler et al. proposed a novel gastroschisis management alternative—the sutureless silo approach (SSA). SSA involves covering the abdominal wall defect with the umbilical cord or a silo to allow sutureless closure with the secondary intention [8]. SSA is also known as the plastic closure and the nonoperative management of gastroschisis. Given that SSA is safe and comparable to the POCS and has potential advantages of better esthetic results, the procedure being transferred from the operative room (OR) to the bedside, and lower costs, SSA has gained wide acceptance in the developed countries and across nations [4, 6, 7, 9–11]. Undoubtedly, the preferred method for the management of gastroschisis has changed fundamentally over time [7]. However, SSA was never performed in Palestine despite its safety and simplicity and the availability of the inexpensive materials. Herein, we report our first-ever case managed with staged abdominal closure using a modified SSA.

## 2. Case Presentation

**Figure 1:**
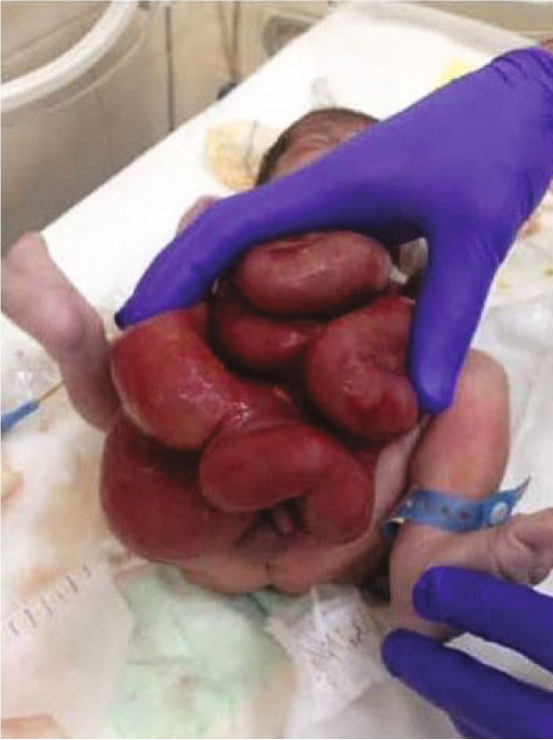
Patient's condition at the delivery.

A currently 18-month-old boy, a product of a full-term vaginal delivery following an uneventful pregnancy with a birth weight of 3000 g, was referred to our neonatal intensive care unit (NICU) due to a ventral abdominal wall defect in the periumbilical region—gastroschisis (Figure 1) at the age of one day. The neonate had an immediate orogastric tube placed and was given intravenous fluid (IVF) expansion with subsequent IVF with antibiotics. Gauzes soaked with warm normal saline were applied around the bowel.

The neonate was transferred to the OR for POCS under general anesthesia. The stomach, transverse and descending colon, and terminal ileum were all outside of the abdominal cavity and dilated without membranous covering. Bowels were warmed using gauzes soaked with warm normal saline with a trial of reduction and primary closure. PCOS failed because the abdominal cavity was too small and the bowels were too swelled. The alternative management was to put the bowels into a silo bag filled with saline and suture the bag to the fascial edges for future repair. Since we did not have the standard silo bag, we used an IV normal saline bag to make a silo.

**Figure 2:**
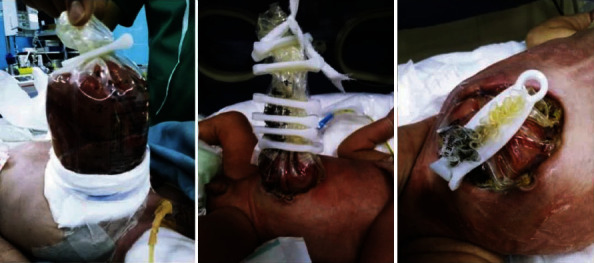
Reduction of the bowels into the abdominal cavity using a silo bag.

**Figure 3:**
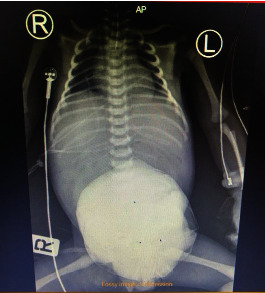
X-ray of the silo bag inside the neonate.

The neonate was connected to mechanical ventilation (MV) and kept nill per os (NPO) postoperatively. An echocardiogram showed a patent foramen ovale, mitral regurgitation, and an evidence of increased pulmonary pressure. Due to the congenital cardiac issues, the infant remained in the NICU for three months. During this time and on subsequent stages, we moved the bowels slowly inside the abdominal cavity and put clamps onto the silo bag to keep bowels in place (Figures 2 and 3).

**Figure 4:**
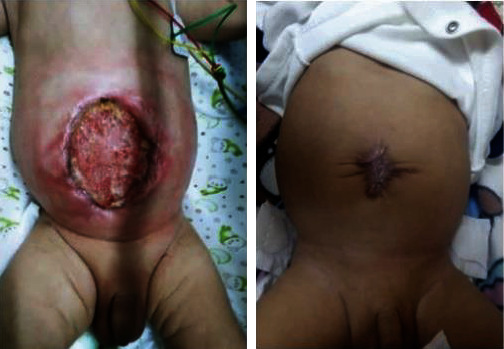
Granulation tissue formation and cosmetic closure of the defect.

Once the bowels were inside, we chose not to close the defect by the delayed primary closure with sutures due to the ongoing cardiac issues. We left the defect opened and covered it with nonadherent dressings for further closure by secondary intention. For the very first time, we saw that the normal skin was adhering to the granulation tissue forming a protective new layer. Therefore, we did not close the defect with sutures. The results of this technique were better than we expected. It made a more cosmetic appearance with a minimally visible scar (Figure 4). When we searched the literature, we discovered this sutureless technique and learned that it is gaining wide acceptance across nations.

**Figure 5:**
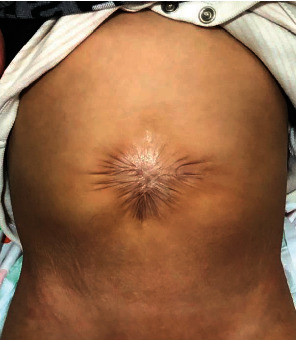
Picture of the abdominal scar after 18 months.

After 3 months managing the coexisted congenital cardiac disease, the infant was able to be disconnected from the MV and reached full feeding capacity. The infant did very well and was discharged home. At routine follow-ups, the infant was gaining weight and doing well. At the age of 18 months, a follow-up showed a normal-appearing child with appropriate length and weight, although with a speech delay. There were no abdominal hernias (Figure 5).

## 3. Discussion

The exact gastroschisis pathogenesis is currently unknown. Several theories have been postulated such as failure of the mesoderm to form the body wall, rupture of the amnion around the umbilical ring, abnormal involution of the right umbilical vein and disruption of the right vitelline artery or yolk sac artery, abnormal body wall folding, gene polymorphisms, and maternal immune response to new paternal antigens [1]. Gastroschisis potential risk factors include young maternal age, cigarette smoking, aspirin use, use of vasoconstrictive and recreational drugs, and maternal genitourinary infections [12]. Gastroschisis incidence rates increased from 0.06–0.8 per 10,000 to 4.5–5.13 per 10,000 in the previous few decades [13].

Gastroschisis is usually detected prenatally on ultrasound by visualizing a paraumbilical abdominal wall defect lacking membranous covering. Otherwise, the diagnosis is made at birth [14].

The immediate management of gastroschisis starts with broad-spectrum antibiotics and fluids to compensate for the large amount of the insensible losses due to the exposed bowels. The bowels should be wrapped with sterile saline dressings covered with a plastic wrap to minimize fluid losses and to preserve body heat. In addition, respiratory support should be provided if needed.

Although many techniques were described for gastroschisis abdominal wall defect repair, all approaches are aimed at getting the bowel back to the abdominal cavity and repairing the fascia and skin. In 1943, Watkins reported the first successful surgical repair: the primary operative closure (POCS) [15]. In 1967, the first staged reduction of the viscera was reported using the Teflon sheets as a silo [16].

PCOS and delayed primary surgery or staged silo closure have remained the mainstay management of gastroschisis. However, these approaches have disadvantages of requiring prolonged intubation and MV, narcotic analgesic use, ileus formation, prolonged hospital stay, and subsequently significant financial burden on both hospitals and families [6, 7].

Therefore, in 2004, a novel management approach known as the sutureless silo approach (SSA) was described [8]. SSA has some reported advantages over the traditional surgery of gastroschisis management. SSA is reported to be safe and comparable to the POCS [4, 6].

SSA provides excellent esthetic results with minimal scar formation [17]. SSA transfers the procedure from the OR to the bedside, thus lowering the financial burden on both hospitals and families [6, 7]. Therefore, it gained a wide acceptance in developed countries and across nations [4, 9–11]. The umbilical cord closure type of SSA without endotracheal intubation and general anesthesia is found to be more successful in smaller, more premature neonates [18]. A study reported that SSA was associated with reduced time needed for extubation, which probably was due to the minimal effect of the sutureless approach on intraabdominal pressure, and the reduced need for narcotics and sedatives. The decreased time needed to extubate gastroschisis patients lowers mechanical ventilation complications [19]. However, a randomized controlled trial showed that SSA is not associated with a significant difference in the length of intubation compared to POCS [6].

Despite the abovementioned advantages of SSA, its safety and simplicity, and the availability of inexpensive materials, SSA was never performed in Palestine. We have managed our first SSA-like case without any knowledge of the presence of such reported and described gastroschisis management approach in the literature. Expectedly, the technique we have used was not identical to the reported technique in the literature. We believe that it is very promising to adopt the SSA in our institution since SSA evidences many advantages and provides reduced hospital stay which is fundamental to our institution due to the limited number of beds and lower management costs to both hospital and families.

A few SSA drawbacks and disadvantages were reported. A study reported an increased incidence of umbilical hernia development in patients with SSA [4]. A randomized controlled trial showed that SSA is associated with a significant increase in time to full feeds and time to discharge [6]; this study may explain why our patient took a long time to reach his full feeds and time to discharge.

## 4. Conclusion

The preferred method for the management of gastroschisis has changed fundamentally over time. The sutureless silo approach (SSA) is a novel approach for gastroschisis management. SSA has advantages over the traditional management options of excellent esthetic results, the procedure being transferred from the OR to the bedside, and the low financial burden on both the hospitals and families. SSA can gain acceptance in the developing countries as it has in developed ones.
